# Impact of individualized opioid protocols on post‐cesarean discharge prescriptions

**DOI:** 10.1002/pmf2.70485

**Published:** 2026-10-30

**Authors:** Sarah W. Freeman, Mohak Mhatre, Megan J. Li, Meghan E. Beard, Leanne Ludwick, Devika Lekshmi, Sebastian Z. Ramos

**Affiliations:** ^1^ Department of Gynecology Lahey Clinic Burlington Massachusetts USA; ^2^ Department of Maternal Fetal Medicine Tufts University School of Medicine Boston Massachusetts USA; ^3^ Department of Internal Medicine George Washington School of Medicine Washington District of Columbia USA; ^4^ Department of Obstetrics and Gynecology Perelman School of Medicine at the University of Pennsylvania Philadelphia Pennsylvania USA

**Keywords:** cesarean delivery, Individualized opioid prescriptions, postoperative pain

## Abstract

**Introduction:**

Consensus on optimal prescribing of opioids at discharge following cesarean delivery remains limited. Since inpatient opioid use is a strong predictor of outpatient need, individualized approaches may improve prescribing accuracy. The optimal individualized prescribing protocol for post‐cesarean pain management at discharge is unknown. While multiple studies have demonstrated benefit with individualized protocols, direct comparison of different protocols has not yet been demonstrated. This study evaluated the modeled application of four individualized prescribing protocols (based on inpatient opioid consumption) on post‐cesarean discharge prescriptions compared to actual prescribing patterns.

**Methods:**

This retrospective cohort study included all patients ≥18 who underwent cesarean delivery from September 1 to December 31, 2023 at a single tertiary academic center. Inpatient opioid use was abstracted from electronic medical records and used to calculate discharge prescriptions based on the following previously published protocols: Bleicher et al. (2024), Osmundson et al. (2018, 2022), and the Prescription After Cesarean Trial (PACT). These modeled prescriptions were compared to the actual prescriptions received at discharge. The primary outcome was total dose prescribed in morphine milligram equivalents (MME); secondary outcomes included number of tablets prescribed and number of patients receiving a prescription at discharge.

**Results:**

A total of 197 patients were included. There were 124 primary cesarean deliveries (63%) and 73 repeat cesarean deliveries (37%). Of these, 186 (94%) received an opioid prescription at discharge and had a median total dose of 75 MME. Total MME prescribed at discharge decreased among >60% of patients using the Bleicher, Osmundson 2022, and PACT protocols, but increased among 64% of patients using the Osmundson 2018 protocol. Compared to actual prescribing, both Bleicher and PACT models reduced the proportion of patients receiving an opioid prescription by 38%, while both Osmundson models resulted in a 6% increase in patients receiving prescriptions.

**Conclusion:**

Within the same population, the impact of individualized post‐cesarean opioid prescribing protocols varied widely. These findings demonstrate that the choice of individualized prescribing model may meaningfully influence discharge prescribing and highlight the need for prospective comparative studies incorporating patient‐reported pain and satisfaction outcomes to identify the optimal approach.

## 1 INTRODUCTION

The US healthcare system is battling an opioid epidemic largely of its own making. In 2023, prescription opioids were responsible for 35 deaths per day [1]. With up to 80% of patients receiving a new opioid prescription following surgery [2, 3], opioid prescribing patterns among surgeons merit particular consideration. Given that post‐cesarean opioid prescriptions constitute the greatest total morphine milligram equivalents (MME) dispensed after surgery in the United States [4], obstetric clinicians are uniquely positioned to critically examine their prescribing practices and potential risks. While fixed‐dose procedure‐based guidelines can provide some assistance, they often lead to under‐ and overprescribing [5, 6]. Additionally, the absence of standardized protocols contributes to substantial variation in prescription quantity and duration across clinicians and geographic regions [7, 8]. Individualized approaches that incorporate inpatient opioid consumption have shown promise in reducing excess prescribing without compromising pain control [9, 10, 11, 12, 13].

In the general surgery literature, multiple studies from Bleicher et al. [10, 14] have demonstrated the functionality of using a simple formula based on inpatient consumption in the 24 h prior to discharge to predict post‐discharge consumption. In the obstetrics literature, Osmundson et al. similarly demonstrated across multiple studies [9, 15] that inpatient opioid use is a strong predictor of outpatient need and can help reduce the number of leftover pills when compared to standard prescribing. More recently, the Prescription After Cesarean Trial (PACT) by Smid et al. [11] demonstrated that prescribing protocols based on inpatient consumption, in conjunction with shared decision‐making, can decrease outpatient prescriptions without compromising pain control.

Current American College of Obstetricians and Gynecologists (ACOG) guidance provides limited direction regarding the optimal quantity of opioids prescribed at discharge, instead emphasizing shared decision‐making and limiting opioid use to the shortest reasonable duration for acute pain [16]. In the absence of consensus, clinicians and institutions are left to develop their own prescribing practices, likely contributing to the substantial variation observed in post‐cesarean opioid prescribing [17, 18]. Emerging evidence supports individualized, data‐driven discharge guidelines based on inpatient opioid consumption [9, 10, 11, 12, 13]. However, existing individualized protocols have not been directly compared within the same patient population, limiting evidence regarding the optimal approach. This study evaluates the modeled impact of four published individualized protocols on post‐cesarean discharge opioid prescriptions compared with actual prescribing patterns at a single academic institution.

## 2 MATERIALS AND METHODS

This is a retrospective cohort study of adults (age ≥18) who underwent cesarean delivery between September 1 and December 31, 2023 at a single tertiary academic center in Boston, Massachusetts. Patients with current or prior substance use, including those on opioid maintenance therapy, were excluded, while those with a history of chronic pain were included. Additional exclusion criteria included concurrent hysterectomy, readmission within 14 days of discharge, transfer to another hospital for neonatal proximity, neonatal demise, and discharge after post‐operative Day 4 (Figure 1).

**FIGURE 1:**
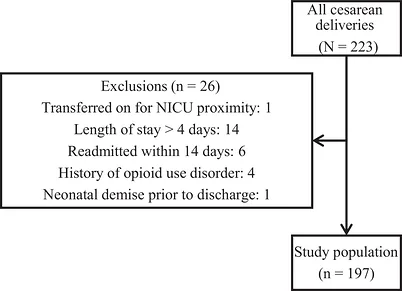
Flow chart of study population and exclusions.

During the study period, no standardized institutional protocol for inpatient pain management or discharge opioid prescribing was in place. Inpatient order sets generally included scheduled intravenous ketorolac for the first 24 h after delivery followed by as‐needed ibuprofen and acetaminophen, although these order sets were not universally used. An Enhanced Recovery After Delivery (ERAD) protocol had not yet been implemented. Discharge opioid prescribing, including prescription quantity, was at the discretion of each individual clinician, with no institutionally specified quantity. Patients receiving opioids at discharge were prescribed either oxycodone 5 mg tablets or hydromorphone 2 mg tablets. Shared decision‐making was not routinely incorporated into discharge opioid prescribing. The Tufts University Institutional Review Board approved this study (IRB#00005162).

### 2.1 Data collection

Data on patient demographics, clinical characteristics, type of anesthesia (spinal, epidural, combined, or general), use of IV pain medication following surgery, and discharge prescriptions were abstracted from the electronic medical record. Additionally, history of depression/anxiety and tobacco use in pregnancy were collected. Use of non‐opioid pain medication (non‐steroidal anti‐inflammatory drugs, Tylenol, and Gabapentin) was also recorded. Neonatal outcomes including neonatal intensive care unit (NICU) admission, stillbirth or neonatal death, and discharge at the time of birthing parent were also collected. Chart abstraction also included new opioid prescriptions, urgent care/emergency room visits, and readmissions within 30 days of discharge. All opioid prescriptions were converted to MME by standard conversion rates for accuracy of comparison [19]. Study data were collected and managed using REDCap. As a quality‐control measure, 10% of charts were independently reviewed by two study team members to verify the accuracy of the data abstraction.

### 2.2 Protocols

The four individualized prescribing protocols evaluated: Bleicher et al. [10], Osmundson et al. [9, 15], and PACT trial [11], were identified from the general surgery and obstetric literature (Appendix 1). These protocols were selected based on their use of inpatient opioid consumption to inform discharge prescribing and their potential applicability to the study population. Table 1 summarizes the characteristics and prescribing approach of each protocol. A more detailed description of the models is included in Appendix 1.

**TABLE 1:** Side‐by‐side comparison of each protocol in comparison to actual with a summary of population, independent variable, and inclusion or exclusion criteria.

Protocol	Actual	Bleicher	Osmundson 2018	Osmundson 2022	PACT
Population^a^	s/p cesarean delivery	s/p open or lsc general surgery admitted for >24 h	s/p cesarean delivery	s/p cesarean delivery	s/p cesarean delivery
Primary variable	N/A	MME consumption 24 h prior to discharge	MME consumption 24–47 h after delivery	MME consumption 24 h prior to discharge	MME consumption 24 h prior to discharge
Additional variables	N/A^b^	None	None	Tobacco use in pregnancy, history of anxiety and/or depression	Shared decision‐making
Inclusion/exclusion criteria					
Concurrent hysterectomy	Excluded	N/A	Excluded	Excluded	Excluded
Substance or opioid use disorder	Excluded	Included	Excluded	Excluded	Excluded
Chronic pain	Included	Included	Included	Excluded	Included
Excluded if readmitted	Within 14 days	Within 20 days	–	–	–
Language requirement	None	English	English or Spanish	English or Spanish	English or Spanish
Minimum prescription at discharge	None	None	48.8 MME	9.8 MME	None

Abbreviations: C/S, cesarean section; LSC, laparoscopic; MME, morphine milligram equivalents; s/p, status post; N/A, not applicable.

^a^ All the studies included only patients 18 years old or older who were opioid tolerant.

^b^ Shared decision‐making component was not evaluated in this study due to the nature of retrospective chart review.

In the general surgery literature, Bleicher et al. [10] have demonstrated the use of a simple prescribing formula based on opioid consumption during the 24 h before discharge, with the recommended discharge prescription calculated as twice the MME consumed during this period. In the obstetric literature, Osmundson et al. [9] demonstrated that individualized prescribing based on inpatient opioid consumption reduced both opioid use and the number of unused tablets after discharge. The Osmundson et al. formula (discharge prescription MME = 48.8 + [1.77 × IP24–47]) uses opioid consumption during 24–47 h after delivery to estimate post‐discharge opioid requirements [9].

Osmundson et al. [15] subsequently developed a multivariable model incorporating opioid consumption during the 24 h before discharge, tobacco use during pregnancy, and a history of depression or anxiety to predict post‐discharge opioid use. We applied this model using the author's publicly available online calculator. Because the calculator accepts only whole number MME values, inpatient MME was rounded to the nearest integer before entry (https://vumc‐chp‐halvorson.shinyapps.io/OpioidUseAfterCes_app/). The calculator accepts a maximum inpatient opioid consumption of 60 MME; therefore, patients with a consumption exceeding 60 MME consumed within 24 h of discharge were included at the capped threshold of 60 MME. In additional analyses, the model was repeated excluding these patients.

Finally, the large, multicenter Maternal Fetal Medicine Units (MFMU) Network randomized controlled PACT trial by Smid et al. [11] combined a prescribing algorithm based on opioid consumption during the 24 h before discharge—assuming a 20% daily decline in opioid requirements—with shared decision‐making regarding the final prescription quantity. Because shared decision‐making was not routinely incorporated into discharge opioid prescribing at our institution, our retrospective application of the PACT protocol was limited to the algorithm‐derived prescription quantity and did not incorporate its shared decision‐making component.

Patients readmitted for more than 12 h within 14 days of discharge were excluded from the analysis due to possible inpatient opioid exposure that could confound assessment of the initial prescription, making it difficult to distinguish opioid requirements related to the initial post‐cesarean recovery from those associated with the readmission. Since 8 days (IQR 6–13 days) [19], has been demonstrated at the median duration of post‐cesarean opioid use, we selected a 14‐day window to encompass the period in which most post‐discharge opioid use occurs. This is also consistent with the Bleicher study [10], which excluded patients who were readmitted within 20 days of discharge.

### 2.3 Data analysis

Patient demographics and clinical details were summarized using descriptive statistics (Table 2). Inpatient opioid consumption was collected for two prespecified intervals and converted to MME: 24–47 h after delivery and 24 h before discharge. Opioid consumption at 24–47 h after delivery was used to calculate modeled discharge prescriptions according to the Osmundson et al. 2018 [9] protocol, while consumption during the 24 h before discharge was used for the Bleicher et al. [10], Osmundson et al. 2022 [15], and PACT [11] protocols. Modeled prescriptions generated by each protocol were compared with actual discharge prescriptions using Wilcoxon sign‐rank test and Hodges–Lehmann estimates for non‐parametric paired differences (Appendix 2). A modeled prescription of 0 MME or 0 tablets, as applicable to the individual protocol, was classified as no opioid prescription. Tablet counts were standardized to oxycodone 5 mg tablets, the institution's standard discharge opioid, to facilitate comparison across protocols. Outcome variables were summarized using descriptive statistics, with nominal variables reported as frequencies and proportions. Continuous variables were reported using median and interquartile range (IQR) and compared using non‐parametric comparison tests as appropriate. *p* values were adjusted for multiple comparisons using Bonferroni's correction. The statistical analysis was performed using Stata Version 19.

**TABLE 2:** Cohort characteristics (*N* = 197).

Characteristic	*n* (%)
Age	
18–24	17 (9)
25–34	111 (56)
35+	69 (35)
Race/ethnicity	
Asian, non‐Hispanic	13 (7)
Black, non‐Hispanic	42 (21)
Hispanic or Latino/Latinx	46 (23)
Other, non‐Hispanic	7 (4)
White, non‐Hispanic	72 (36)
Unknown or not reported	17 (9)
Insurance	
Private	86 (44)
Public	111 (56)
Primary language	
English	151 (77)
Spanish	19 (10)
Haitian Creole	11 (6)
Portuguese	7 (4)
Other	9 (5)
Body mass index at delivery	
18.5–24.9	14 (7)
25–29.9	52 (26)
30–34.9	52 (26)
35 or higher	79 (40)
Gestational age	
< 28 weeks	4 (2)
28w0d–33w6d	19 (10)
34w0d–36w6d	25 (13)
37w0d–40w6d	140 (71)
≥41 weeks	9 (5)
Increased risk of opioid dependence	
History of chronic pain	6 (3)
History of anxiety/depression	72 (36)
Tobacco use in pregnancy	7 (4)
Number of cesarean(s)	
Primary	124 (63)
Second C/S	44 (22)
Third C/S	18 (9)
Fourth C/S	11 (6)
Maternal length of stay after delivery	
2 days	19 (10)
3 days	130 (66)
4 days	48 (24)
Neonatal outcomes	
NICU admission	54 (27)
Discharged same day as birthing parent	161 (82)
Neonatal demise or stillbirth	1 (< 1)
Complications within 30 days of discharge	
Readmission	2 (1)
New opioid prescription	5 (2)
Urgent care or emergency room visit	15 (8)
Readmit, new prescription, or ED/UC visit	19 (10)

*Note*: Categorical variables are presented as frequency (%), percentages within categories that do not sum to 100% are due to rounding.

Abbreviations: C/S, cesarean section; ED, emergency department; NICU, neonatal intensive care unit; UC, urgent care.

## 3 RESULTS

Of the 223 eligible individuals, 197 were included in the final analysis (Figure 1). Exclusions included discharge after postoperative Day 4 (*n* = 14), transfer to another institution for neonatal proximity (*n* = 1), readmission for >12 h within 14 days of discharge (*n* = 6), history of substance use disorder or maintenance therapy for opioid use disorder (*n* = 4), and neonatal death (*n* = 1). Baseline demographics and clinical characteristics are presented in Table 2. Of the included patients, 124 (63%) underwent primary cesarean delivery and 73 (37%) underwent repeat cesarean delivery. A history of chronic pain was present in 3% of patients while 36% had a history of anxiety or depression. Median maternal length of stay was 3 days. No cesarean hysterectomies occurred. For neonatal outcomes, 54 (27%) of neonates were admitted to the NICU. Most neonates, 161 (82%), were discharged home on the same day as their birthing parent.

Of the 197 patients included, 186 (94%) received an opioid prescription at discharge. The median total dose prescribed was 75 MME (IQR 45–75; Table 3). The median discharge prescription was ten 5 mg oxycodone tablets (IQR6‐10) with a range of 0–20 tablets. Application of all four individualized prescribing protocols decreased both median MME and the number of tablets prescribed compared with actual prescribing (Table 3), but varied in their impacts within individuals.

**TABLE 3:** Magnitude and relative change in discharge MME and 5 mg oxycodone tablets across individuals by protocols in comparison to actual prescriptions (*N* = 197).

Protocol	Overall median (IQR)	Median paired difference (95% CI)^a^	Relative change	*p* value^b^
Total Morphine Milligram Equivalents				
Actual	*75.0 (45.0–75.0)*	*Ref*	*Ref*	–
Bleicher	15.0 (0.0–45.0)	−37.5 (−41.3, −30.0)	−50%	<0.001
Osmundson et al. (2018) [9]	66.5 (48.8–84.2)	+7.9 (4.8, 11.3)	+10%	<0.001
Osmundson et al. (2022) [15]	32.1 (9.8–64.4)	−18.2 (−21.9, −13.2)	−24%	<0.001
PACT	22.5 (0.0–97.5)	−11.3 (−22.5, −3.8)	−15%	0.039
Total 5 mg Oxycodone Tablet Count				
Actual	*10 (6–10)*	*Ref*	*Ref*	–
Bleicher	2 (0–6)	−5 (−6, −4.)	−50%	<0.001
Osmundson et al. (2018) [9]	9 (7–12)	+1.5 (+1, +2)	+15%	<0.001
Osmundson et al. (2022) [15]	5 (2–9)	−1.5 (−2, −1)	−15%	<0.001
PACT	3 (0–13)	−1.5 (−3, −1)	−15%	0.012

Abbreviations: CI, confidence interval; MME, morphine milligram equivalents; Ref, reference data from actual prescriptions.

^a^ Data derived from Hodges–Lehmann estimate showing absolute difference between modeled and actual prescriptions for individual patients, shown as median (confidence interval).

^b^ *p* values from Wilcoxon sign ranked test, adjusted for multiple comparisons.

The Osmundson et al. 2018 [9] protocol demonstrated a median increase of 7.9 MME (Table 3, *p* < 0.001), with 64% of patients modeling a higher dose and tablet count than they had received at discharge (Table 4, *p* < 0.001). Conversely, the Bleicher et al. [10], Osmundson et al. 2022 [15], and PACT protocols resulted in ≥ 60% of patients receiving less MME and tablets at discharge (Table 4). The magnitude of these adjustments varied by protocol (Table 3), with a relative decrease of 50%, or 5 less tablets, using the Bleicher protocol (*p* < 0.001), and a more modest relative decrease of 15%, or 1.5 less tablets, using both the PACT (*p* = 0.012) and Osmundson et al. [15] protocols (*p* < 0.001).

**TABLE 4:** Direction of change in discharge opioid prescriptions by protocol in comparison to actual prescriptions (*N* = 197).

Protocols	Decreased MME	Increased MME	No change	*p* value^a^
Bleicher	156 (79%)	20 (10%)	21 (11%)	<0.001
Osmundson et al. (2018) [9]	71 (36%)	126 (64%)	0 (0%)	<0.001
Osmundson et al. (2022) [15]	131 (67%)	66 (33%)	0 (0%)	<0.001
PACT	121 (61%)	65 (33%)	11 (6%)	0.039

Protocols	Decreased tablets	Increased tablets	No change	*p* value^a^
Bleicher	155 (79%)	21 (11%)	21(11%)	<0.001
Osmundson et al. (2018) [9]	58 (29%)	127 (64%)	12 (6%)	<0.001
Osmundson et al. (2022) [15]	126 (64%)	66 (34%)	5 (2%)	<0.001
PACT	121(61%)	64 (32%)	12 (6%)	0.012

*Note*: Data are *n* (%); tablets refer to oxycodone 5 mg tablets.

Abbreviation: MME, morphine milligram equivalents.

^a^ *p* values from Wilcoxon sign ranked test, adjusted for multiple comparisons.

The protocols also differed in the proportion of patients who would have received any opioid prescription. The Bleicher and PACT protocols reduced the proportion receiving an opioid prescription by 36%; under either protocol, 42% of patients would have been discharged without an opioid prescription (Table 5), compared with 6% under actual prescribing. In contrast, both Osmundson protocols generated an opioid prescription for every patient, increasing the proportion receiving an opioid prescription from 94% under actual prescribing to 100% under either Osmundson protocol.

**TABLE 5:** Comparison of opioid discharge prescriptions sent across all protocols (*N* = 197).

	Actual	Bleicher	Osmundson 2018	Osmundson 2022	PACT
Discharged with Rx	187 (94%)	114 (58%)	197 (100%)	197 (100%)	114 (58%)
		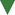 36%	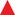 6%	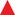 6%	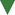 36%

*Note*: Data are *n* (%); 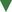 or 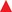 denotes increase or decrease in comparison to actual prescriptions.

Abbreviation: Rx, Prescription.

In the 30 days following discharge, five patients (2.5%) required an additional opioid prescription for pain management. All five patients would have received an opioid prescription under each of the four modeled protocols. Four patients had opioid consumption exceeding the 60 MME maximum accepted by the Osmundson et al. [15] calculator. In the primary analysis, values exceeding 60 MME were capped at 60 MME. Additional analysis excluding these patients yielded no change in median MME or tablet count; therefore, these patients were retained in the primary analysis.

## 4 DISCUSSION

This study demonstrates that individualized protocols based on inpatient consumption have the potential to substantially alter the quantity of opioids prescribed at discharge after cesarean delivery, although the direction and magnitude of impact vary by protocol. Therefore, protocol selection matters. Under the Bleicher and PACT protocols, 42% of patients would have been discharged without an opioid prescription, compared with only 6% under actual prescribing. This practice is more consistent with prescribing patterns in Europe [20, 21], where rates of opioid use disorder and opioid‐related deaths are significantly lower than those in the United States [22, 23, 24]. These differences demonstrate that individualized prescribing protocols are not interchangeable and suggest that protocol selection may meaningfully influence opioid exposure at discharge. Direct prospective comparison is needed to determine which approach best balances opioid reduction with adequate post‐discharge pain control.

The decrease in MME and total tablets prescribed across protocols in this study differed in magnitude from previously published work. Osmundson et al. 2018 [9] demonstrated a 35% decrease in MME and tablet count in their study, whereas our results demonstrated a median increase of 15% in both MME and tablet counts. The decrease in the overall median in the Osmundson 2018 model despite a trend toward increased dosing at an individual level is due to significantly skewed data (Appendix 2). These differences may also reflect the comparatively restrictive baseline prescribing practices at our institution. More than 75% of patients in our cohort were prescribed 10 or fewer oxycodone 5 mg tablets at discharge, compared with 30 tablets in the control group of Osmundson et al. 2018 [9] and 20 tablets in the PACT trial, suggesting that individualized prescribing may have more varied impacts in restrictive prescribing practices. The PACT trial did not report MME but observed a 30% reduction in the median number of tablets prescribed in the individualized prescribing group as compared to a 70% decrease in median tablets in our study. Notably, when shared decision‐making was incorporated, patients frequently chose to receive more opioids than recommended by the prescribing algorithm. Because shared decision‐making could not be reproduced in our retrospective study, our application of the PACT model may overestimate the reduction in opioid prescribing that would occur with implementation of the complete PACT intervention.

While this study does not specifically address prescription adequacy, the original studies of Bleicher et al. [10], Osmundson et al. 2018 [9], and the PACT trial [11] demonstrated no significant differences in prescription adequacy or patient‐reported pain outcomes between standardized and individualized prescribing approaches. In these studies, requests for additional prescriptions and the proportion of prescribed opioids consumed were similar between control and intervention groups, despite differences in initial prescription quantity. These findings align with prior evidence suggesting that prescribing smaller quantities of opioids may reduce overall consumption, as opioid use can be influenced by factors beyond pain alone, including patients’ perceptions that they should finish prescribed medication or a desire to retain unused medication for future pain [3, 11]. Additionally, in our cohort, all patients who required additional prescriptions after discharge had initially received an opioid prescription and would have received one under each of the four modeled protocols, although the adequacy of these prescriptions cannot be evaluated in this retrospective study. Furthermore, the low rate of additional prescriptions should not be interpreted as a sign of prescription adequacy given the inability to capture prescriptions received outside our health system.

Notably, the Osmundson et al. 2022 [15] model was developed to predict post‐discharge opioid consumption using inpatient clinical characteristics but has not been prospectively evaluated as a discharge prescribing intervention. Therefore, unlike the other three protocols, its effects on prescription adequacy and patient satisfaction have not been evaluated prospectively. In addition, three patients in our cohort had inpatient opioid consumption exceeding the 60 MME maximum accepted by the publicly available Osmundson et al. [15] calculator, two of whom did not have a history of chronic pain. Although additional analysis excluding these patients did not alter median MME or tablet count, the calculator's upper input limit may restrict its application to patients who consume more than 60 MME during the 24 h before discharge.

This study has several strengths. First, it leverages detailed medical record data to obtain precise inpatient opioid consumption, allowing for accurate modeling of individualized prescriptions. Second, the use of four previously published protocols allows for direct comparison of how different individualized prescribing approaches perform within the same patient population and clinical setting, providing greater insight into their practical differences. Additionally, the inclusion of patients with chronic pain who were not receiving chronic opioid therapy increases the applicability of these findings to patients for whom postpartum pain management may be more challenging. Finally, the study addresses a clinically meaningful gap by quantifying differences in prescribing recommendations across individualized protocols and the potential magnitude of opioid reduction achievable with each approach.

This study also has several limitations. First, as a retrospective analysis, it is subject to potential inaccuracies and incomplete documentation inherent to electronic medical records. Shared decision‐making could not be reproduced retrospectively, precluding complete application of the PACT intervention and potentially overestimating the reduction in opioid prescribing that would occur with its implementation. The study was conducted at a single academic institution over a 4‐month period, which may limit generalizability to other practice settings. In addition, an ERAD protocol had not been implemented during the study period, potentially limiting generalizability to institutions with standardized multimodal analgesia protocols already in place. Because no standardized protocol for inpatient pain management or discharge prescribing existed during the study period, inter‐clinician variability may have influenced observed prescribing patterns. Although opioid doses were standardized using MME, these conversions are approximate and may not reflect true clinical equivalence. Post‐discharge outcomes including opioid refills or emergency visits occurring outside our health system may not have been captured, potentially underestimating subsequent opioid use or inadequate pain control. Finally, this study did not directly assess patient satisfaction, pain scores, or outpatient opioid consumption, limiting our ability to assess prescription adequacy.

Our findings have important clinical and societal implications. The estimated economic burden of opioid use disorder and fatal overdose exceeded one trillion US dollars in 2017 [25], and opioid‐related mortality continues to reduce US life expectancy, with disproportionate effects among Black, Hispanic, and Native American populations [26]. These harms underscore the need for evidence‐based prescribing strategies that minimize unnecessary opioid exposure and leftover medication while maintaining adequate postoperative analgesia. Although the opioid crisis is multifactorial and cannot be addressed through a single intervention, individualized prescribing represents one potentially actionable strategy. Given the high prevalence of cesarean delivery, even modest improvements in tailoring postoperative opioid prescriptions could translate into meaningful, population‐level reductions in opioid exposure. Our findings further suggest that the specific individualized protocol selected matters, underscoring the need for prospective comparative evaluation to determine which approach best balances opioid stewardship with adequate pain control.

## 5 CONCLUSION

In summary, individualized prescribing protocols based on inpatient opioid use can substantially reduce modeled opioid quantities prescribed at discharge after cesarean delivery, although this is not universally true in all protocols across all populations. Additionally, the magnitude of reduction and likelihood of receiving an opioid prescription vary by protocol. These differences highlight the need to determine which prescribing approach offers the greatest clinical utility while maintaining prescription adequacy. A prospective, comparative evaluation of opioid prescribing strategies incorporating patient‐reported pain scores, functional recovery, and assessment of unused medication would provide valuable insight into balancing adequate pain control with harm reduction. Ultimately, evidence‐based, standardized prescribing protocols may help optimize post‐cesarean pain management while minimizing excess opioid exposure.

## AUTHOR CONTRIBUTIONS

**Sarah W. Freeman**: Conceptualization; investigation; writing—original draft; writing—review and editing; data curation; project administration; methodology; visualization; validation. **Mohak Mhatre**: Conceptualization; investigation; methodology; supervision; formal analysis; project administration; writing—review and editing; resources. **Megan J. Li**: Data curation; writing—review and editing; validation. **Meghan E. Beard**: Validation; writing—review and editing; data curation. **Leanne Ludwick**: Validation; writing—review and editing; data curation. **Devika Lekshmi**: Formal analysis; supervision; software; methodology; validation. **Sebastian Z. Ramos**: Conceptualization; investigation; writing—review and editing; visualization; methodology; validation; formal analysis; supervision.

## CONFLICT OF INTEREST STATEMENT

The authors declare no conflicts of interest.

## ETHICS STATEMENT

The study was approved on July 15, 2024, and monitored by the Institutional Review Board at Tufts University Medical Center (IRB#00005162).

## Figures and Tables

**FIGURE A2 pmf270485-fig-0002:**
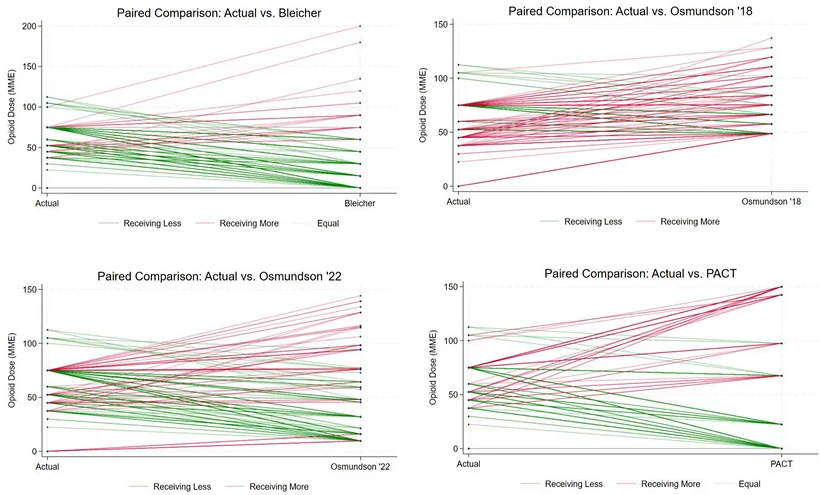
Paired profile plots showing MME change across individuals from actual prescribing to each modeled protocol (*N* = 197).

## Data Availability

The data that support the findings of this study are available upon request from the corresponding author upon reasonable request. The data are not publicly available due to privacy or ethical restrictions. On request, de‐identified clinical data are available. The prescribing protocols used in the theoretical analyses are included in Appendix 1.

## APPENDIX 1

### 1.1

The formulas from each of the original studies are demonstrated below. The morphine milligram equivalents (MME) for each theoretical discharge prescription was calculated using the input of MME consumed during one of two pre‐specified intervals: 24–47 h after delivery or 24 h prior to discharge. Pill calculations were made under the assumption of prescribing 5 mg oxycodone tablets.

24PDC = MME consumed in the 24 h prior to discharge
24‐47PD = MME consumed between 24 and 47 h after delivery
DP = MME prescribed at discharge

**BLEICHER FORMULA**

$$\begin{array}{ccc} \mathrm{DP} & = & 24\mathrm{PDC}\times 2\\ \mathrm{number}\;\mathrm{of}\;\mathrm{pills} & = & \left\lceil \frac{24\mathrm{PDC}\times \;2}{7.5}\right\rceil \end{array}$$

**OSMUNDSON 2018 FORMULA**

$$\begin{array}{ccc} \mathrm{DP} & = & \left(24-47PD\times 1.77\right)+48.8\\ \mathrm{number}\;\mathrm{of}\;\mathrm{pills} & = & \left\lceil \frac{\left(24-47PD\times 1.77\right)+48.8}{7.5}\right\rceil \end{array}$$

**OSMUNDSON 2022 FORMULA**

The complete mathematical formula is not publicly available; however, the calculator can be found at https://vumc‐chp‐halvorson.shinyapps.io/OpioidUseAfterCes_app/.

**PACT FORMULA** a The PACT trial included a shared decision‐making component in which patients in the intervention group were presented with the calculation as above and then permitted to select a number between 0 and 20 pills. As this study was a retrospective chart review, this component was not included.

$$\begin{array}{ccc} \mathrm{DP} & = & 4\left\{\begin{array}{c} \left(0.8\;\times \;7.5\right)=6\;÷7.5\;∼\;1\\ \left(0.8\;\times \;6\right)=4.84÷7.5\;∼\;1\\ \left(0.8\;\times \;4.8\right)=3.8÷7.5\;∼\;1\\ \left(0.8\;\times \;3.8\right)=3.1÷7.5\;∼\;1 \end{array}\right.\\ \mathrm{DP} & = & \left\lfloor \log_{0.8}\left(\;\frac{3.75}{24\mathrm{PDC}}\right)\right\rfloor \\ \mathrm{number}\;\mathrm{of}\;\mathrm{pills} & = & \sum_{k=0}^{N}\left\lfloor \frac{0.8^{k}\times \;24\mathrm{PDC}}{7.5}+0.5\right\rfloor \end{array}$$

## APPENDIX 2

### 2.1

[Fig pmf270485-fig-0002]
